# Neural Network for Nanoscience Scanning Electron Microscope Image Recognition

**DOI:** 10.1038/s41598-017-13565-z

**Published:** 2017-10-16

**Authors:** Mohammad Hadi Modarres, Rossella Aversa, Stefano Cozzini, Regina Ciancio, Angelo Leto, Giuseppe Piero Brandino

**Affiliations:** 1 Institute for Manufacturing, Department of Engineering, University of Cambridge, 17 Charles Babbage Road, Cambridge, CB3 0FS United Kingdom; 2 CNR-IOM Istituto di Officina dei Materiali c/o SISSA, via Bonomea 265, 34136 Trieste, Italy; 3 eXact-Lab srl, via Beirut 2, 34151 Trieste, Italy; 4 CNR-IOM, TASC Laboratory, Area Science Park, Basovizza S.S. 14 km 163.5, Trieste, 34149 Italy; 5 Elegans.io Ltd, Bellside House 4th Floor, 4 Elthorne Road, London, N19 4AG United Kingdom

## Abstract

In this paper we applied transfer learning techniques for image recognition, automatic categorization, and labeling of nanoscience images obtained by scanning electron microscope (SEM). Roughly 20,000 SEM images were manually classified into 10 categories to form a labeled training set, which can be used as a reference set for future applications of deep learning enhanced algorithms in the nanoscience domain. The categories chosen spanned the range of 0-Dimensional (0D) objects such as particles, 1D nanowires and fibres, 2D films and coated surfaces, and 3D patterned surfaces such as pillars. The training set was used to retrain on the SEM dataset and to compare many convolutional neural network models (Inception-v3, Inception-v4, ResNet). We obtained compatible results by performing a feature extraction of the different models on the same dataset. We performed additional analysis of the classifier on a second test set to further investigate the results both on particular cases and from a statistical point of view. Our algorithm was able to successfully classify around 90% of a test dataset consisting of SEM images, while reduced accuracy was found in the case of images at the boundary between two categories or containing elements of multiple categories. In these cases, the image classification did not identify a predominant category with a high score. We used the statistical outcomes from testing to deploy a semi-automatic workflow able to classify and label images generated by the SEM. Finally, a separate training was performed to determine the volume fraction of coherently aligned nanowires in SEM images. The results were compared with what was obtained using the Local Gradient Orientation method. This example demonstrates the versatility and the potential of transfer learning to address specific tasks of interest in nanoscience applications.

## Introduction

Image classification, the task of giving an input image a label from a set of categories, is an active and quickly evolving research area. Image recognition, image retrieval and other algorithms based on deep learning are now widely applied in many different areas, from classification of electrocardiograms from the human heart^1^ and classification of lung cancer^2^ to even happiness estimation^3^.

The ability to identify and recognise specific features within images is of particular interest to scientists working with microscopy techniques. Neural networks have been employed for machine learning in a number of recent studies, extracting features from different types of microscope images. For example, recognition of cellular organisms from scanning probe microscopy images was shown using artificial neural networks^4^. Chowdhury *et al*.^5^ demonstrated the ability to recognize specific microstructural features of interest, such as dendrites, from micrographs of varying magnifications using pre-trained convolutional neural networks for feature extraction. In the study by Al-Khedher *et al*.^6^ the morphology of carbon nanotube structures, such as their curvature and alignment, was estimated using a neural network classifier.

Image recognition techniques can be a very powerful tool in nanoscience, where a large number of images are the typical outcome of characterization techniques such as scanning electron microscopy. A scanning electron microscope (SEM) is a versatile instrument which is routinely used in nanoscience and nanotechnology to explore the structure of materials with spatial resolution down to 1 nm. Advances in SEM technology, particularly the complete computer control of the instrument and the increased ease of operation, have enabled users to independently operate the microscope and to acquire images with limited training^7^. In most research and industrial centers, SEMs work as multi-user facilities, and generate large numbers of images per user and per field of application. However, in spite of the wide range of scientific contexts in which SEM is employed, there are not appropriate tools for classifying and archiving the large amount of data generated. Currently, SEM images are stored in the computer control units and labelled according to personal users’ criteria, that strongly limit their long-term exploitation as well as reproducibility, due to the lack of a well-defined classification paradigm.

One of the key challenges in scientific research including nanoscience is the long-term management of data produced. Recently, a clear set of principles guiding this process has been published: data should be Findable, Accessible, Interoperable and Reusable (FAIR Guiding Principles^8^). Such an approach favors the idea that sharable data will validate findings, will promote their reuse, and will stimulate collaborations.

Therefore, it is evident that data warehouse and data mining tools which extend and complement image metadata must be designed for effective data sharing. This is one of the main objectives of the Nanoscience Foundries & Fine Analysis NFFA-Europe project. NFFA-Europe brings together twenty European nanoscience research laboratories, half of which are co-located with Large Scale Facilities, with the aim of providing users from both academia and industry with open access to advanced instrumentation and theory. NFFA offers a single entry point for proposal submission, and a common platform named IDRP (Information and Data management Repository Platform) to support access and integration of the resulting experimental data. The main goal of the IDRP development is to semi-automatize the harvesting of scientific data generated by many different instruments available at the NFFA-Europe sites, and of their metadata to allow them to be searchable according to the FAIR Guiding Principles. Metadata design is part of a joint research activity within NFFA-Europe that takes empirical input from the project participants, relying on state-of the art standards and practices. Such efforts led to a published metadata design for nanoscience^9^.

Within the NFFA-Europe experimental facilities, the SEM is one of the core characterization tools, available at ten sites. As the starting point of our work and the subject of this paper, we focused on the SEM available at the CNR-IOM (Istituto di Officina dei Materiali) located in Trieste. This instrument features a dataset of more than 150,000 images, the result of more than five years of experiments performed by dozens of users. It therefore represents a relevant case to test the performance of automatic tools for image recognition to store, classify, and finally make available such a considerable amount of data on the IDRP.

Training a neural network on SEM images would provide many advantages to nanoscience researchers: (i) automatic image classification, avoiding the need for the users to classify each recorded image; (ii) a searchable database which allows scientists to find a specific category of SEM images; (iii) potential for feature extraction to accomplish specific tasks, as will be discussed in more detail further on.

Common machine learning algorithms traditionally address isolated tasks and rely on large amounts of training data. Transfer learning aims to overcome this limitation by developing methods to transfer knowledge learned from one task to another^10^. In the context of neural networks, the feature extraction technique enables the application of learned features of a network, trained on a large dataset, on a new problem, by freezing the parameters of all but the last layers, and retraining only them.

One of the most commonly used datasets is ImageNet^11^, which has been gathered over many years, and is publicly available. Models trained on ImageNet seem to capture details that are generally relevant in any image classification task.

Usually, lower layers of convolutional neural networks capture low-level image features, e.g. edges, while higher layers capture more complex details. The final layers are generally assumed to capture information that is relevant for solving the specific task, image classification in our case. While the initial layers of the pre-trained network can be frozen, the last few layers must be trained to learn the specific features of the new dataset. Feature extraction usually results in faster computing times than training a new convolutional neural network from scratch, because not all the parameters need to be estimated.

In this work, we mainly focused on the feature extraction technique, by retraining the final layers of an Inception-v3^12^ deep convolutional neural network, implemented with the TensorFlow (TF) library^13^, to perform the classification task on SEM images. Such results have been complemented with additional tests using other models, implementations, and methods. All the SEM images have been provided by the CNR-IOM institute in Trieste.

## Results and Discussion

In this section, we will present the scientific results achieved through the transfer learning approach we propose. These results address the initial part of the ambitious work we are doing within the NFFA-Europe project to provide the nanoscience community with a searchable database of SEM images. The incremental steps performed in this work are the following:

1. Define a training set, which means agree upon the most suitable criteria to classify the SEM images and provide a label for each of them. It is worth specifying that this is the major outcome of our work, soon to be published as an individual result. This represents the first specific training set for SEM images, which can be used as a reference set for future deep learning applications in the nanoscience domain;
2. Apply the transfer learning approach on the SEM dataset. This task comprises two subtasks:
  - Feature extraction from an Inception-v3 model pre-trained on the ImageNet 2012 dataset, by retraining the last layers (softmax + fully connected layer) on the SEM dataset;
  - Compare the results in terms of test accuracy obtained with the standard TF implementation of the Inception-v3 model with the ones provided by the TF-slim^14^ new implementation, namely Inception-slim, and with other recent models (Inception-v4 and Inception-ResNet-v2^15^);
3. Employ the image recognition algorithm to label the test SEM images with the categories they are supposed to belong to (together with the probability score), and collect statistics on the obtained results to calibrate the level of automation in a classification workflow;
4. Benchmark the performance of the classification workflow on a HPC cluster when processing a massive amount of SEM images (from thousands to hundred of thousands);
5. Illustrate an example where the transfer learning technique can provide novel insights in nanoscience research, and compare the results with the ones obtained by an existing method.

### Defining the training set

All our work is based on a supervised learning task, thus a set of training data labelled by humans must be provided. This section explains the process by which SEM images were classified by the authors.

For the training to be effective, a significant number of images for each category should be provided and, most importantly, the images should be a good representation of what the learning algorithm will be asked to recognize. Due to the highly diverse nature of SEM users, images fall in a wide span of different classes. The categories were determined by taking into account visual pattern characteristics of the images rather than more abstract classes related to domain specific notions. However, where possible, the classification was chosen such that the categories would be related to the work of the scientists, and close to the way how they might tag or label the images themselves.

One way of classifying nanostructures based on their shape and structure is their dimensionality, which is visually distinct and easy to be classified by the neural network, as well as a relevant property, since many of the functionalities of the nanostructures are strongly related to it. We can classify nanostructures as 0D, 1D, 2D, and 3D objects. In this classification, 0D objects refer to particles which can be dispersed and isolated over an area of the sample, or clustered together. 1D objects are often referred to as nanowires, rods, or fibres. These structures are often packed together, to form bundles, for instance, or aligned parallel to each another and along a specific direction (i.e., growth direction). 2D structures refer to films and coatings on a surface, which can be formed from a variety of different materials with a range of surface topologies: some surfaces can seem smooth and flat on a SEM, whilst others are made of small particles packed together, covering the entire surface. 3D structures can refer to pillars, or other devices like Micro Electro-Mechanical Systems (MEMS), typically fabricated using lithographic processes. After further investigation of SEM images, additional categories such as biological samples and tips were added in order to fully cover the spectrum of images in our database.

**Figure 1:**
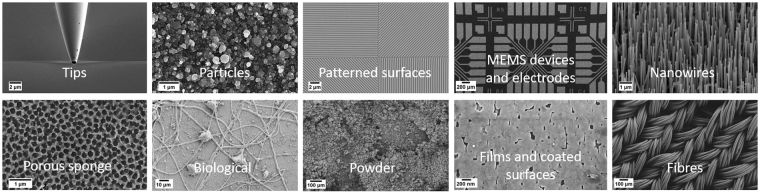
Categories chosen for SEM images. The dimensionality of nanoscience objects provided the basis for the choice. Other categories, such as Biological and Tips were added as these were common images found in the SEM database.

The final choice encompasses a set of ten categories for a total of 18,577 images, shown in Fig. 1. The breakdown of the number of images in each category is shown in Table 1. It has to be noted that three categories have a smaller number of images: the impact of such categories on the overall performance of the trained network will be discussed and explained in the next sections. These ten categories cover a broad range of SEM images, and are representative of many different areas of nanoscience. The ongoing classification was presented by authors to several nanoscientists and SEM users, and was then discussed together. This final set is thus considered representative enough of the wide area of SEM images. However, as will be discussed later, depending on the magnification chosen, certain categories can look very similar to one another, posing considerable challenges for the image classification.

**Table 1:** SEM training set.

Tag	Category	N images
L0	Porous_Sponge	171
L1	Patterned_surface	3310
L2	Particles	3412
L3	Films_Coated_Surface	308
L4	Powder	895
L5	Tips	1561
L6	Nanowires	3656
L7	Biological	953
L8	MEMS_devices_and_electrodes	4158
L9	Fibres	153
	TOTAL	18577

### Achievements on Transfer Learning

#### Inception-v3 feature extraction

Initially, we retrained the last layer of the Inception-v3 model, pre-trained on the ImageNet 2012^11^ dataset, using the SEM training set defined above. When retraining the neural network, a portion of the images is automatically kept aside by the algorithm for validation and testing. We evaluated the performance of the algorithm by reporting the test accuracy (the proportion of total correct guesses with respect to the total test set), the precision (the fraction of retrieved instances that are relevant), and the recall@1 (the fraction of relevant instances that are retrieved) after 4,000 iterations.

Our feature extraction algorithm splits the total sample of images as suggested by Haykin^16^: 80% as the main training set, 10% as validation set, and 10% as test set.

The feature extraction algorithm with our adopted distribution is able to reach an average test accuracy of ~90%, a precision of ~80%, and a recall@1 of ~90% on a test set containing ~10% of the total sample (1853 unseen images). For this case, we also report in Table 2 the confusion matrix. The number of correct and incorrect predictions are broken down and summarized as percentage values with respect to the total number of images in each category. The diagonal elements in the confusion matrix represent the number of correctly classified images of each category, i.e. the number of test images with a certain ground truth label that actually obtained the same label during classification, whilst the off-diagonal elements represent classification errors, i.e. the number of images that ended up in another category during classification. Looking at Table 2, it can be noted that some categories, like Porous_Sponge, Films_Coated_Surface, and Fibres, are recognized very effectively, even though they are the least populated ones. This shows that the low number of images in these categories does not negatively affect the performance of the algorithm, probably because their features are sufficiently clear and distinct to be easily recognized by the network. On the other hand, the predictions for some categories like Patterned_surface and MEMS_devices_and_electrodes appear more scattered. We will further investigate the reason of this outcome in the image classification section.

**Table 2:** Confusion matrix with the prediction (expressed in percentage) for each category obtained when testing the feature extraction task.

		Predicted labels										N images
		L0	L1	L2	L3	L4	L5	L6	L7	L8	L9	
True labels	L0	90.91	0.00	0.00	4.55	4.55	0.00	0.00	0.00	0.00	0.00	22
	L1	0.00	79.38	4.52	1.13	0.85	3.11	1.69	0.00	8.19	1.13	354
	L2	0.58	0.29	94.74	1.75	1.17	0.58	0.29	0.29	0.29	0.00	342
	L3	0.00	0.00	3.33	90.00	3.33	0.00	0.00	3.33	0.00	0.00	30
	L4	2.44	2.44	1.22	3.66	85.37	0.00	1.22	3.66	0.00	0.00	82
	L5	0.72	3.60	3.60	0.72	0.00	87.05	0.72	0.72	2.88	0.00	139
	L6	0.26	1.56	1.82	2.08	1.56	2.34	87.53	1.82	0.78	0.26	385
	L7	1.22	0.00	1.22	2.44	0.00	0.00	2.44	92.68	0.00	0.00	82
	L8	0.25	5.43	0.49	0.74	0.25	1.73	1.23	0.49	89.38	0.00	405
	L9	0.00	8.33	0.00	0.00	0.00	0.00	0.00	0.00	0.00	91.67	12
	TOTAL NUMBER OF IMAGES											1853

The categories are reported with their tag Ln, n = [0, 9] for shortness. As reported in Table 1, the mapping is the following: L0 = Porous_Sponge, L1 = Patterned_surface, L2 = Particles, L3 = Films_Coated_Surface, L4 = Powder, L5 = Tips, L6 = Nanowires, L7 = Biological, L8 = MEMS_devices_and_electrodes, L9 = Fibres.

#### Comparison with other models

The recent release of a new abstraction library (TF-slim^14^) over TF allowed us to carry out further tests, with the goal of validating the results achieved in the previous section and eventually improving the performance. In this section, we present the results obtained by using TF-slim for different experiments on the same set of images.

First, we retrained our network using the TF-slim implementation of Inception-v3 in the same way we did for the previous version: we started from the checkpoint trained until convergence on the ImageNet 2012 dataset, provided by the TF team, and we trained the final layers with the weights initialized to zero. We will refer to this model as Inception-slim. Subsequently, we also compared the performance of Inception-v4 and Inception-ResNet-v2 (ResNet for shortness) models^15^, when retraining the last layer as we did for Inception-v3 and Inception-slim.

For each experiment, we ran 5,000 training steps on the same sample of SEM images, and then evaluated the models. The test accuracies, averaged over 5 runs, are reported in column 2 of Table 3. The recognition performance of Inception-slim, as expected, gives similar results to the previous TF implementation, when using the same set of configuration parameters. The performance of Inception-v4 and ResNet are comparable, as first shown by Szegedy *et al*.^15^, although ResNet accuracy is slightly worse compared to the others. This is because this models seems to train slower, thus is still rather unstable at 5,000 steps, as reflected by the big error bar in column 2 of Table 3.

**Table 3:** Results after 5,000 iterations when retraining the last layers using different models.

Model	Accuracy [%]	Computing time [s]
Inception-v3	89.8 ± 0.4	434 ± 9
Inception-slim	88.9 ± 0.6	3600 ± 20
Inception-v4	89.2 ± 0.3	6135 ± 25
ResNet	87.5 ± 1.1	7018 ± 58

The test accuracy has been averaged over 5 runs, and the error reported is the standard deviation.

We also compared the performance in terms of computing time for all the models we used, reported in column 3 of Table 3. It can be immediately noted that Inception-v3 is remarkably faster than the other models: 5,000 iterations ran in ~7 minutes, against ~1 hour for Inception-slim and ~2 hours for Inception-v4 and ResNet, on the same hardware node equipped with 2 GPUs. This is because in Inception-v3 the bottlenecks (see the Methods and Tools section for details) were cached, while in the other implementations all the weights up to the last layers had to be retrieved for each image every time it was reused during training.

### Achievements on image classification

As already illustrated, the confusion matrix in Table 2 helps in inspecting the performance of our adopted algorithm. In this section, we want to shed light not only on which types of images the neural network found more challenging to categorize, but most of all on the reason why they were problematic. This is required to understand whether the loss of performance on those categories is due to the training itself or reflects a challenge for the neural network, intrinsic in some types of images.

To reach this goal, we selected a second test set of 1068 unseen images, distributed as in Table 4. In this test set, the most scattered categories (see Table 2) were split into sub categories which represented the types of image found. Some of these subcategories might look more similar to images in other categories, and so the classification could be less effective for them. All the subcategories have been examined by performing the classification on a representative sample of images, and the results were analyzed. Finally, we performed some statistics on the global image classification task and summarized the results.

**Table 4:** The breakdown of the second test set. For each category, the number of images (N image) and of subcategories (N sub) is also reported.

Category	N image	N sub
Tips	120	3
Nanowires	170	5
Fibres	40	1
Biological	53	1
MEMS_devices_and_electrodes	96	5
Patterned_Surfaces	271	12
Particles	100	4
Powder	70	2
Films_Coated_Surfaces	135	3
Porous_Sponge	13	1

Moreover, some images showed elements pertaining to multiple categories. They have been kept separate from the training sample, and included in the second test set. Then, the classification performance on them has been investigated as a further separate test.

#### Image classification of subcategories

In this section, an explanation is provided for why an incorrect category has scored highly for a set of test images which should all belong to another category. According to the confusion matrix (Table 2), the categories with the most dispersed outcomes (Tips, Nanowires, MEMS_devices_and_electrodes, Patterned_surfaces, and Particles) were not the ones with the fewest training examples. Thus, we can assert that the relatively poorer classification performance in these cases was not due to the small number of examples in the training set, but to the features themselves. By inspecting the SEM images, we noticed that at high magnification some images of different categories becomes almost undistinguishable, and the classification cannot be as effective for such images.

**Figure 2:**
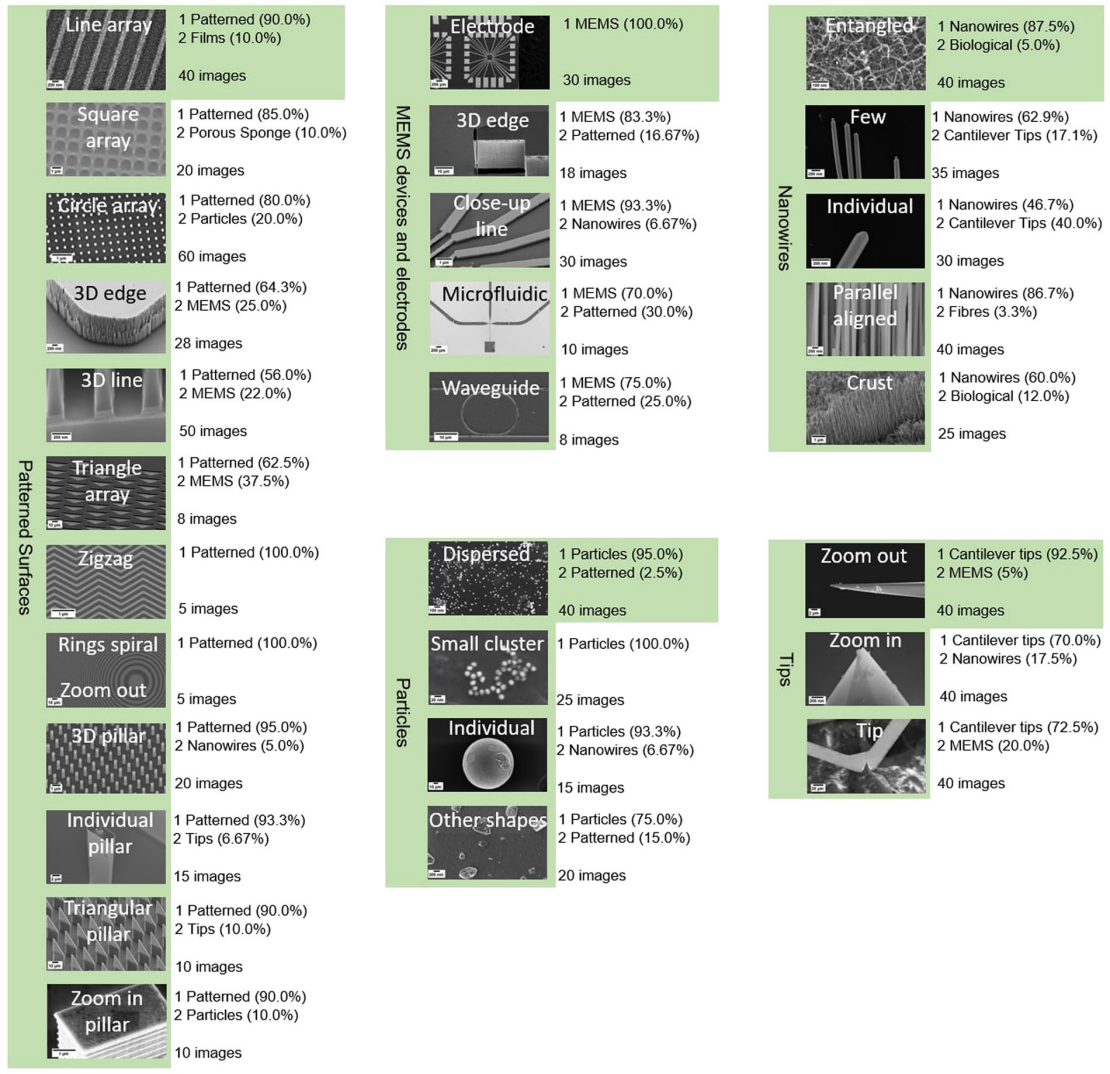
A summary of the image classification outcomes for the most scattered categories. For each subcategory, the two categories which were top ranked the highest proportion of time are shown. The percentage after each category refers to the percentage of times that category was top ranked. The subcategory with the coloured box was deemed to be the most representative of that category as a whole.

A summary of the classification results is shown in Fig. 2, in which the results for the two categories which were top ranked the highest proportion of time have been shown. The subcategory with the coloured box was deemed to be the most representative of that category as a whole. It can be seen that the most representative subcategory was correctly identified ~90% of the time for the majority of the categories.

The Tips category was split into three subcategories, zoom_out images in which most of the length of the tip can be seen, zoom_in images where only the very end of the tip is seen, and tip_on_cantilever which are tips sitting at the end of a cantilever structure, often imaged at a distance such that the cantilever is the dominant feature. The image recognition was most effective on zoom_out images, which are the representative images of Tips. Cantilevers are also found in certain MEMS devices, which explains why MEMS_devices_and_electrodes was the top ranked category in 20% of images in the Tip_on_cantilever test folder. It is important to remark that such results strictly rely on the intrinsic features occurring in our test set.

Entangled_nanowires were correctly classified in 87.5% of cases. However, when Few or Individual nanowires were tested, they also scored highly for Tips. Looking at the images of zoom_out tips in Fig. 2, we can see by eye that there is also a close resemblance between these subcategories, and so this is not a surprising result.

Image recognition was very successful for MEMS_devices_and_electrodes. When the classification was incorrect, the top-ranked category was Patterned_surfaces. This is expected since MEMS devices are usually fabricated using the same technique as patterned surfaces, and they share many features in common, notably smooth edges of structures made from top down processes such as lithography. For the same reason, many images in the Patterned_surfaces category were classified as MEMS_devices_and_electrodes. Moreover, since the Particle category has been strongly identified with dispersed circular dots, it is unsurprising that 20% of Circle_array images had this category as top-ranked.

Particles were well identified in almost all subcategories. However, performance could be improved for non-circular particle shapes by including more images of this type in the training set.

#### Images within multiple categories

481 images showed elements of two categories within the same image. These were kept separate from the train sample, and subsequently processed through the image classification. As expected, the neural network was able to identify both the categories within the images, with often the dominant category receiving a higher score, as the following examples demonstrate.

**Figure 3:**
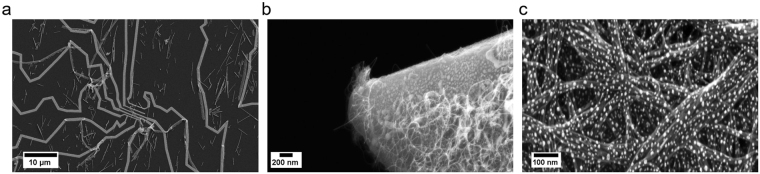
An example of images within two categories. A representative image of (**a**) MEMS electrodes covered by nanowires; (**b**) a tip covered by nanowires; (**c**) nanowires decorated with particles along their length.

Some MEMS electrodes had nanowires dispersed over them (Fig. 3a). The dominant feature in these images was the electrode like structure, since the nanowires were mostly dispersed and isolated from one another. These observations are reflected in the scores, with MEMS_devices_and_electrodes being the top ranked category in 69.2% of cases, whilst for 23% of images Nanowires were identified as the top ranked category. Nanowires were sometimes present at the very end of tips (Fig. 3b). In these cases, 56% of images were classified as Nanowires, and 28% as Tips. In some cases, nanowires had small particles on their surface along their length (Fig. 3c). If these images had to be classified into one category, they would be classed as Nanowires, reflecting the dominant feature, whilst the particles are decorating their surface. This is reflected by the behaviour of the image classification, which selected Nanowires as the correct category in over 85% of cases, and Particles as the correct category in ~9% of the cases.

#### Statistics on image classification

To perform a statistical analysis of the results obtained from the image classification, we processed the 1068 images in the second test set. Each category was assigned a score between 0 and 1, which represents the probability assigned by the algorithm that the image belongs to that category. We focused on the top two predicted labels obtained as output, together with their respective scores. By comparing the predicted labels with the ground truth ones, we could distinguish three possible cases:

- the top ranked category was the correct one;
- the second ranked category was the correct one;
- Both the top two categories were incorrect.

**Figure 4:**
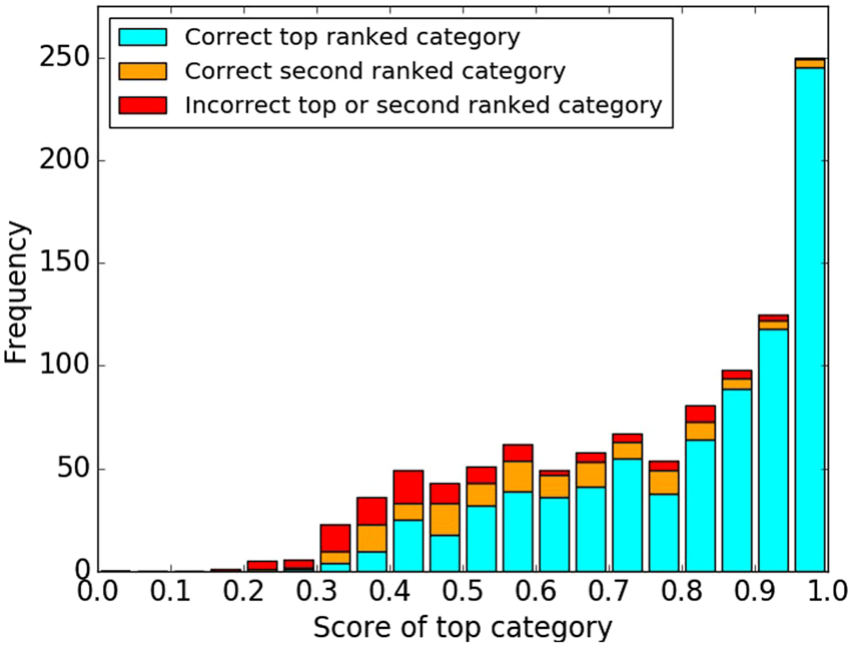
A histogram of the score of the top ranked category. Either the top ranked category was correct (cyan), or it was incorrect with the second ranked category being correct (orange), or incorrect with neither the second ranked category being correct (red).

A histogram of the obtained results is shown in Fig. 4. It can be noted that when the score of the top ranked category is high (e.g. above 0.8), there is a high frequency for that category to be correct. However, when the score is low (e.g. below 0.5), it is more likely that the top ranked category is incorrect. This matches with our intuitive understanding: if the classifier is confident that an image belongs to a certain category, it will assign that category a score close to 1; otherwise, it will assign a low score even to the top ranked category.

**Figure 5:**
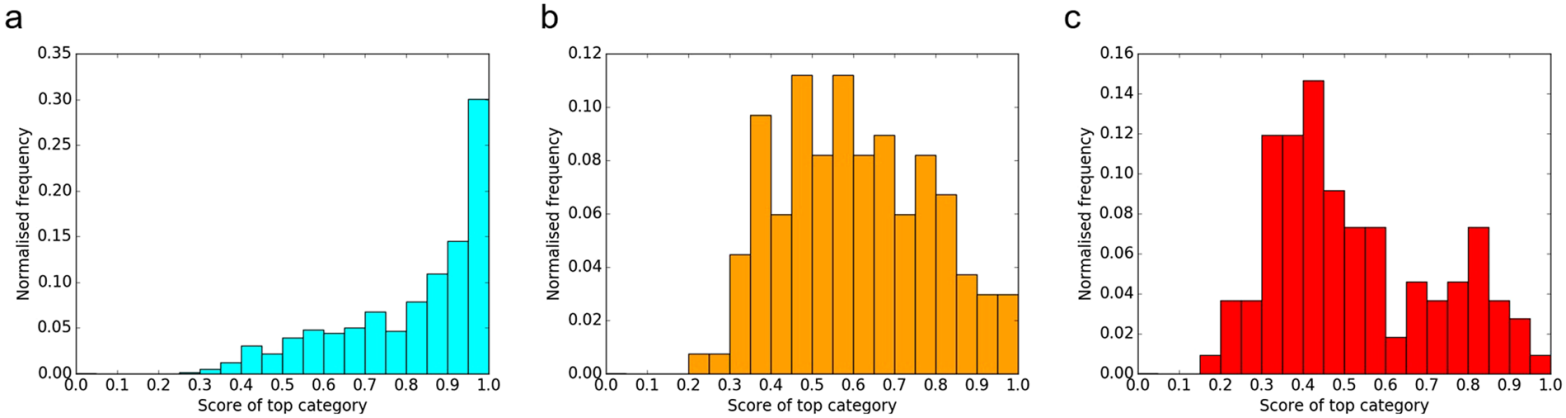
The normalized distribution of scores for each of the groups. (**a**) Top category score when the top category was correct; (**b**) Top category score when the second ranked category was correct; (**c**) Top category score when neither of the top two categories were correct.

This concept can be further clarified by focusing on the distribution of normalized frequencies for each of the three cases, shown in Fig. 5. It can be seen that the peak of the distribution shifts from a score of 0.95-1 when the top ranked category was correct, to 0.5–0.6 when the second ranked category was correct, to 0.4 when neither the first or the second category were correct.

**Figure 6:**
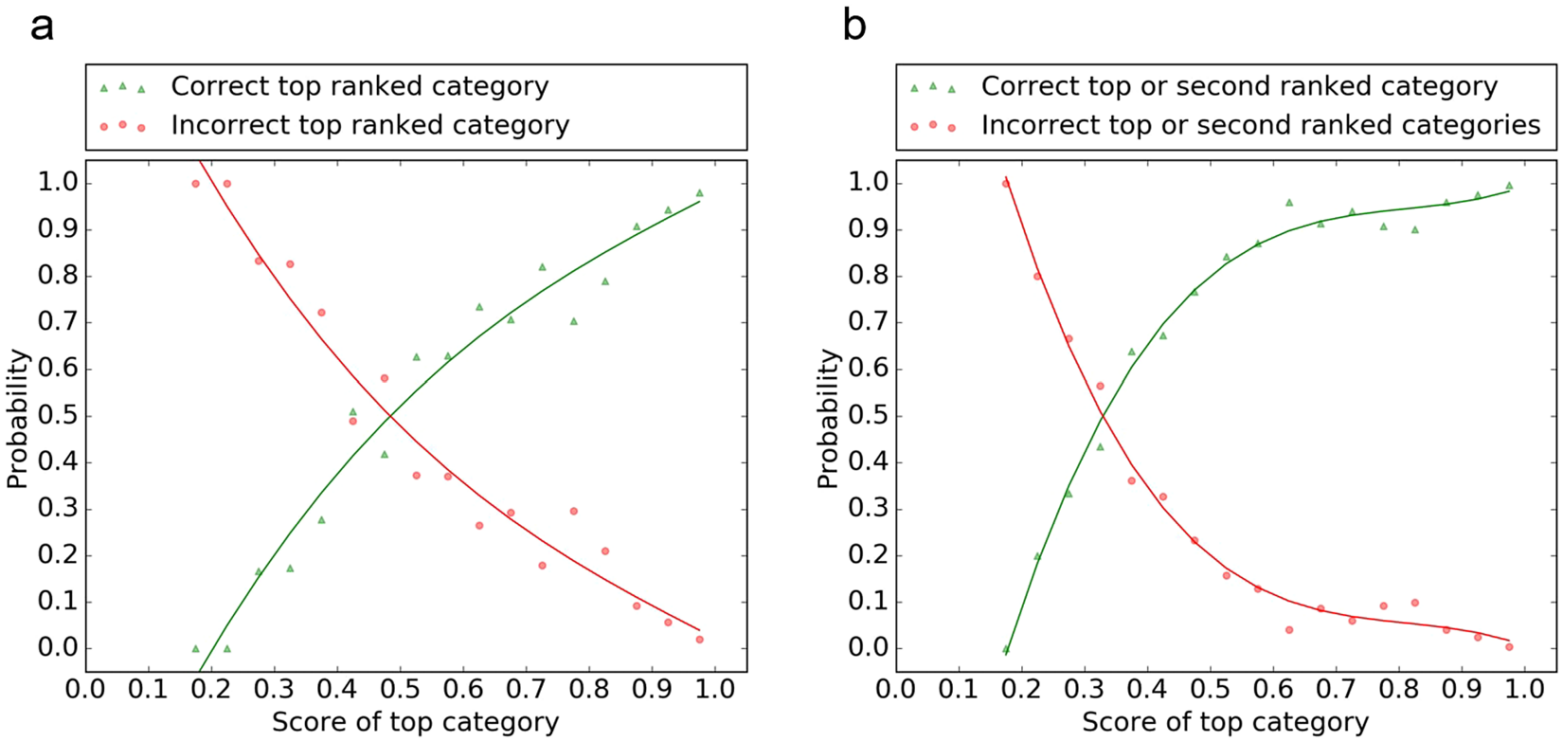
Probability that the top ranked score is assigned to the correct (green) or incorrect (red) category. Given the score of the top category: (**a**) probability that it is assigned to the correct category; (**b**) probability that either the top or the second category is assigned to the correct category. In each case the inverse is also plotted, with the crossover point showing the score at which the two scenarios are equally likely.

The relative frequency of the top ranked score was used to calculate the probability that a given top ranked score was assigned the correct category. Figure 6a shows that this probability, plotted in green, increases for higher scores. The inverse, i.e., the probability that a given top ranked score is assigned to an incorrect category, is plotted in red. The crossover of lines indicates the point at which the two cases are equally likely (i.e., probability = 0.5). In Fig. 6a, it can be seen that when the top ranked score is 0.5, there is 50% chance that the assigned category is correct. Figure 6b shows the probability that, for a given top ranked score, the correct category is either the first or the second prediction (green), and its inverse, i.e., the probability that both predictions are incorrect (red). In this case, the crossover occurs at a lower score: when the top ranked score is 0.35, there is a 50% chance that the correct category is within the top two predictions.

These results can be used to calibrate the level of automation in a workflow which processes the images produced by the SEM instrument, classifies them, and labels them by including the predicted category as an additional scientific metadatum. Figure 4 suggests that when the first score is high (≳0.8) it may be sufficient to automatically assign the first category with a high confidence of it being the correct one. Figure 6 shows that the top two ranked categories could be suggested to the user if the top ranked score is low (between 0.8 and 0.35). For lower first scores (≲0.35), the user will need to choose among more categories. In any case, we can be confident that, even when the top ranked category is not correct, in more than half of the cases (55%) the second top ranked category is correct.

#### Massive image processing

As the last part of this work, once the appropriate thresholds were defined, the image classification algorithm based on TF library has been deployed and adapted to be able to run in parallel on the whole batch of images (roughly 150,000) provided by CNR-IOM. The massive image processing has been done on an Apache Spark cluster deployed through Docker containers on our High Performance Computing infrastructure. The parallel implementation allowed us to study the performance of the whole program on a large number of images.

**Figure 7:**
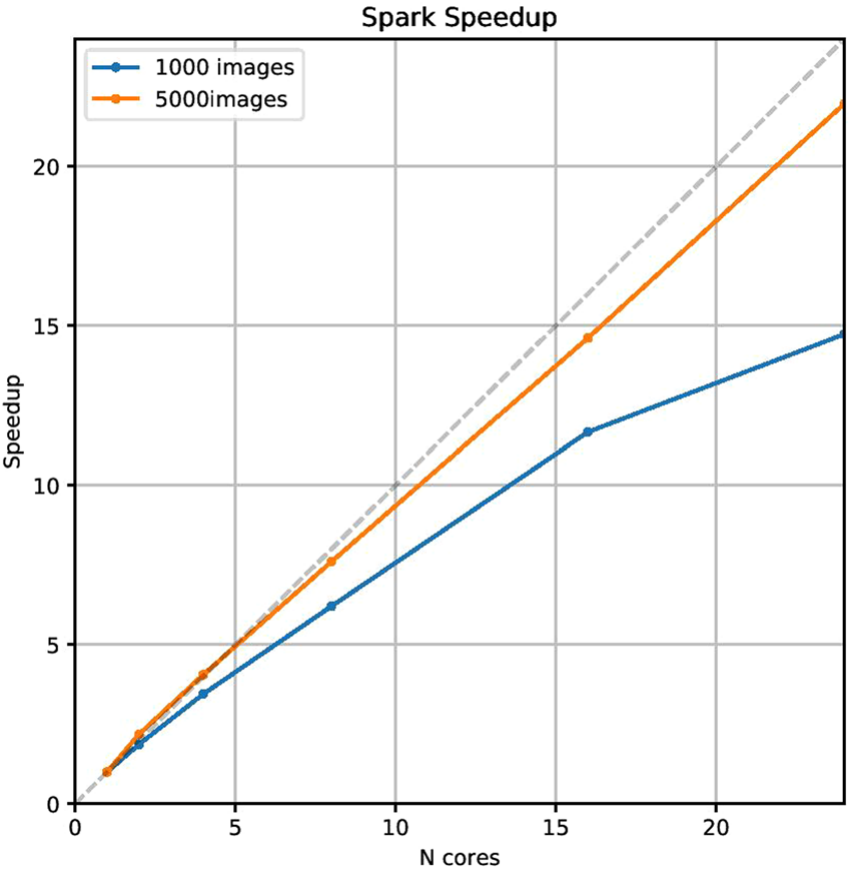
Speedup of our Spark implementation as a function of the number of cores on the cluster node, using a sample of 1,000 (blue) and 5,000 (orange) images. Images were located in a Lustre file system. Errorbars, of the order of 20%, are not reported to avoid confusion.

As a preliminary but illustrative result, we show in Fig. 7 the speedup (the ratio between the execution time on multiple cores and the execution time on single core) of the image classification algorithm in our Spark implementation, as a function of the number of cores within a single node when 1,000 (blue), and 5,000 (orange) images were processed in parallel. The images were stored on a high-performance parallel Lustre file system. The scalability of our application on the sample with 1,000 images was almost linear up to 4 cores, and generally good up to 16 cores; when more resources were employed, the speedup got worse: this could be due to the combined effect of overwhelmed capacity of the file system and increased communication among processes. When processing a sample with 5,000 images, we have seen remarkable improvements in the speedup, which was linear up to 8 cores and still outstanding up to all the 24 cores available. The higher the number of images, the better the scaling, because the communication time becomes a less relevant fraction of the total execution time.

To conclude this section, we mention that 150 and 75 computational hours were used to process all the 150,000 SEM images on 24 and 48 cores, respectively. Further and more detailed investigations, as well as a comparison among different file systems, will be afforded in a forthcoming paper.

### A nanoscience task: mutually coherent alignment of nanowires

A further investigation was performed to determine whether transfer learning can address issues more advanced than only image classification. In particular, we used the feature extraction technique to quantify the fraction of coherent alignment in SEM images of nanowires.

Morphology, homogeneity and orientation of nanoscale objects are among the most relevant issues to be addressed to realise nanomaterials with targeted properties. In particular, 1D nanostructures such as nanowires are used in a diverse range of applications from photonics to energy devices^17,18^. Factors such as the length, diameter, number density and orientation of the nanowires can determine the performance of such devices^19,20^.

The possibility of extracting information on the relative fraction of oriented nanowires entangled within highly dense bundles from a SEM image is challenging, and provides a useful complement to the standard information which is possible to obtain by SEM analysis.

Alignment of nanowires in a SEM image is determined by their orientation relative to a reference direction. Aligned nanowires are oriented in the same direction with a low spread in angular distribution, whilst non-aligned nanowires have random directions with no prominent common orientation.

Alignment can be determined through the manual measurement of the orientation of individual nanowires, by tracing the 1D nanostructures as a straight line and measuring the angle with respect to a reference direction. Due to the large number of nanowires in individual images, this method is time consuming and tedious. Thus, alternative image processing methods have been developed^21,22^. For example, the Fast Fourier Transform method has been used to identify the periodic behaviour in an image^20,23^. The local gradient method, which is a particular case of regression method, has been used to quantify orientation and isotropy properties of a region of interest in an image^24^, based on the evaluation of the structure tensor in a local neighborhood^25^. Special tools have been developed for image analysis packages, such as OrientationJ plugin for ImageJ^26^.

In this work, we approached the problem by retraining the last layers of the Inception-v3 model on just two nanowire categories, labelled either aligned (114 images) or not-aligned (128 images). The images were chosen such that the aligned category had the best possible coherent alignment, and the not-aligned category had a completely random distribution of nanowires, i.e. zero coherent alignment. Subsequently, a test set of 299 images with different degrees of alignment were classified, and the outcomes were collected.

**Figure 8:**
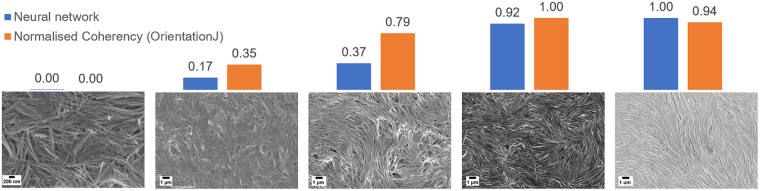
Comparison of alignment score from the image classification obtained by a trained neural network (blue bars) and from the OrientationJ plugin (orange bars) on representative nanowires test images. As the nanowires becomes more mutually aligned, their score approaches 1. Local regions of mutual alignment or of disorder in the image, are detected by the OrientationJ plugin, causing disagreement between the two methods in some cases.

Our results showed that the network is best suited in identifying highly aligned from not-aligned images, as expected since it was trained on a binary choice. However, for images which were in between the two extremes, scores for alignment between 0 and 1 were obtained, and these reflected the degree of ordering of the nanowires in the images, and thus can be used as a measure of their alignment. Some images and their scores are shown in Fig. 8. The scores for the aligned category by image classification are compared with a normalised value for alignment (coherency) using the OrientationJ plugin on ImageJ. The closest agreement is for images with scores close to 0 or 1, with greater divergence for images with scores in between. In this case, it is worth noticing that sometimes the two results disagree because our method focuses on a global alignment, while the OrientationJ plugin targets local mutually aligned regions in the image.

The advantage of using our trained network is that a large batch of SEM images can be *automatically* classified based on the degree of ordering of the enclosed 1D nanostructures, allowing the user to perform a first selection of the most relevant cases in a fully automated way. Subsequently, further more detailed analysis on the selected images can be performed by image recognition methods. This property will be handled as an additional searchable metadatum to be included in the SEM database. This showcases the potential for transfer learning to be used for addressing other challenges in nanoscience.

## Conclusions

In this work, we presented a complete approach to automatically classify nanoscience SEM images by means of transfer learning technique and in particular feature extraction on deep convolutional neural networks. The main outcomes of this work are the following:

• We created and manually annotated the first sample of classified SEM images (for a total of 18,577 images). This dataset will be published as a standalone result, which can be used as a set for future applications of deep learning enhanced algorithms in the nanoscience domain. A second set (1068 images) with a further splitting in subcategories has also been created for testing purposes. The classification criterion was primarily based on the dimensionality of the nano- or micro-scale objects represented in the images. The following ten categories were identified and populated: Particles, Fibres, Biological, Patterned_surface, Nanowires, Tips, Films_Coated_Surface, Porous_sponge, MEMS_devices_and_electrodes, and Powder.

• We investigated the transfer learning technique on the above dataset using different implementations of the most recently used deep convolutional networks:

1. We retrained the last layers of the Inception-v3 model. The average test of the algorithm achieved ~90% accuracy, ~80% precision, and over ~90% recall@1;
2. We found compatible results when comparing the performance obtained by retraining the last layers of different architctures on the same image dataset;

• We tested the image classification algorithm on a second test set to further investigate the results both on particular cases and from a statistical point of view.

1. Our results demonstrate the image classification to be highly effective in detecting distinct and representative images of each category. However, due to the broad range of magnification allowed by the SEM, some objects were difficult to classify. On the other hand, data augmentation techniques could be exploited to artificially increase the number of images in our SEM dataset, especially in the least populated categories, to further increase the training performance;
2. We used the statistical outcomes on the collected test data to calibrate the implementation of our image classification algorithm in a typical NFFA workflow. Our results indicate that the classification of unseen images is correct more than 80% of the time when the top rank score is >0.8. Thus, this rank may be sufficient to automatically assign the first category with a high confidence of it being the correct one. For a lower first rank score, the most likely categories could be suggested to the user to choose from;
3. The image classification algorithm used to obtain the results presented above has been implemented to process hundreds of thousands of images in parallel. A preliminary benchmark result of our Spark implementation processing up to 5,000 images in parallel was shown, and the computing time needed to process all the 150,000 images was also provided.

• The versatility of transfer learning was also demonstrated by extracting features from the Inception-v3 model on SEM images showing different bundles of nanowires, to quantify the volume fraction of coherently aligned nanowires in each image. To validate our results, we compared them with the ones obtained using a more commonly adopted regression method, in particular the Local Gradient Method. This example shows that the feature extraction task can be tailored to address specific tasks of interest in nanoscience applications.

The semi-automatic classification process we developed includes the outcoming labels as additional metadata in the images generated by the SEM, with the final purpose of creating a searchable database for nanoscience.

This work, bridging the field of machine learning and nanostructural characterization by SEM, witnesses the first application of a complete semi-automatic workflow to process data outcomes. Our approach paves the way towards the implementation of new methods and tools which can be applied to a wide range of nanoscience use cases and suitably tuned to resolve specific features of nanomaterials.

## Methods and Tools

We used the C3E Cloud Computing Environment managed by eXact-lab srl. The cluster node is equipped with two Intel Xeon CPUs E5-2697 at 2.70 GHz (12 cores each, for a total of 24 cores) and two K20s Nvidia GPUs. To exploit the resources of the computational node, our Spark cluster has been configured by running a Docker driver with 1 CPU, and two Docker workers with 12 CPUs each.

The algorithm uses the filename of the images to determine which set they should belong to, according to the testing percentage and validation percentage, and returns a dictionary containing an entry for each label subfolder, with images split into training, validation and testing sets within each label. This is designed to ensure that images do not get moved between sets on different runs, since it could generate overfitting problems if images that had been used for training a model were subsequently used in a validation set. To decide the appropriate set and to keep existing image files in the same set even if more images are subsequently added, a hash of the file is used to generate a probability value.

The first phase of the algorithm analyzes all the provided images and calculates the *bottleneck* values for each of them. Bottleneck is an informal term often used for the layer just before the final output layer that actually does the classification. This penultimate layer, which is a meaningful and compact summary of the images, has been trained to output a set of values used by the classifier to distinguish between all the categories it has been asked to recognize. Calculating each bottleneck takes a significant amount of time. Since every image is reused multiple times during training, bottlenecks values are cached on disk so they do not have to be repeatedly recalculated.

Once the bottlecks are completed, the actual training of the top layer of the network begins. Each step randomly takes a batch of images from the training set, finds their bottlenecks from the cache, and feeds them into the final layer to get predictions, which are then compared against the ground truth labels to update the final layer’s weights though the back-propagation process. Finally, evaluation is performed on the test set. The algorithm writes out a version of the Inception-v3 network with the retrained final layer to a GraphDef protocol buffer and the labels to a text file. These two files are the only two elements needed for the SEM image classification algorithm.

We explored the performance of the learning process varying the training steps from 2000 to 10000. Doubling the number of steps, doubled the time, as expected, but the average accuracy did not increase significantly, despite the network having been trained for twice as long, because the model converged very rapidly.

To perform image classification, we adapted the classify_image.py Python script available on the TF GitHub page^27^. The algorithm creates a graph from the GraphDef protocol buffer we obtained from the retraining. Then it runs inference on an input jpg or png image using the last layer of the retrained network and the list of labels. Finally, it outputs a human readable string of the predictions along with their probabilities.

### Data availability

SEM images included in this work are property of CNR-IOM. Their use is only restricted to the purpose of the current study which relates to machine learning and image recognition, and not for any other scientific scope related to their content. For further information refer to Regina Ciancio on behalf of CNR-IOM.
